# Supplementary figures and images for: User review analysis of dating apps based on text mining
Source: PLoS One. 2023 Apr 26;18(4):e0283896. doi: 10.1371/journal.pone.0283896 (PMC10132638; doi:10.1371/journal.pone.0283896)

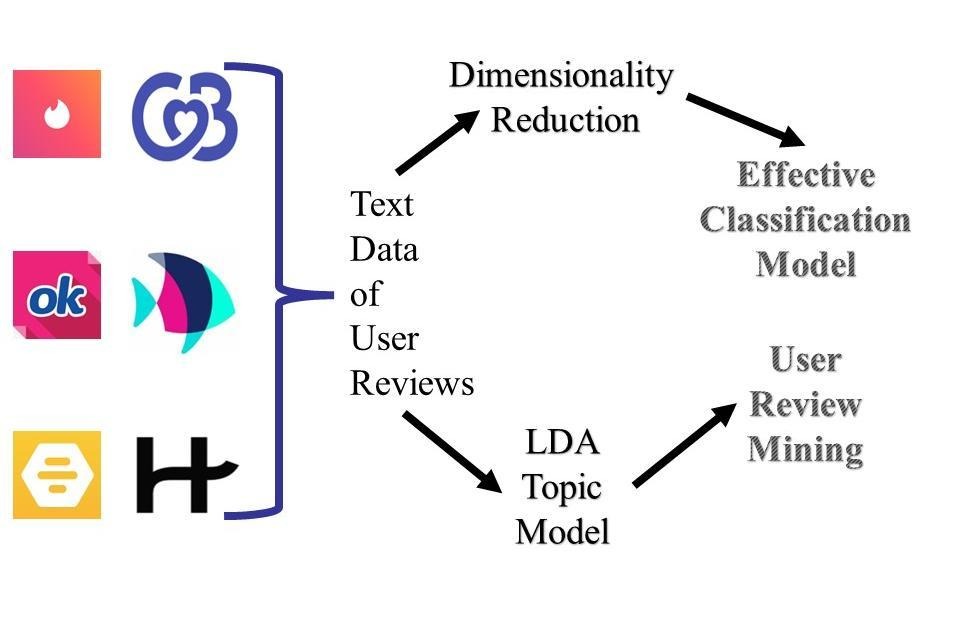

Supplement: S1 Graphical abstract — (TIF) [file pone.0283896.s001.tif]
